# Primary health care physicians’ prescribing patterns for children under five in Qassim, Saudi Arabia

**DOI:** 10.1017/S1463423619000148

**Published:** 2019-06-25

**Authors:** Saulat Jahan, Abdullah Mohammed Al-Saigul, Salih Ahmed Hamdelsseed

**Affiliations:** 1 Public Health Specialist, Research and Information Unit, Public Health Administration, Qassim, Saudi Arabia; 2 Chief, Research and Information Unit, Public Health Administration, Qassim, Saudi Arabia; 3 Technical Supervisor, Buraidah Sector, Public Health Administration, Qassim, Saudi Arabia

**Keywords:** drug prescribing, primary care physician, primary health care, Saudi Arabia

## Abstract

**Background:**

Irrational prescription of drugs in children is reported to be widespread. There are scarce studies on the pediatric prescribing pattern especially at primary health care (PHC) level.

**Aim:**

To determine the physicians’ prescribing patterns for children under five years, to explore completeness of prescriptions’ recorded information, and to analyze the core indicators of drug prescribing at primary health care centers (PHCC) in Qassim.

**Methods:**

This cross-sectional study was conducted on 25 randomly selected PHCCs. All prescriptions, for the first week of first six months of the year 1437 Hijrah (October 2015 to April 2016), were reviewed. Among 25 012 prescriptions, 4125 (16.5%) were for children under five years. We randomly selected 1212 prescriptions for the study. World Health Organization (WHO) specified drug use indicators, and index of rational drug prescribing (IRDP) with a maximum value of 5, were calculated. The physicians and pharmacists of sampled PHCCs were also surveyed to explore prescribing issues.

**Findings:**

The completeness of recorded date, patient age, and gender was more than 90%. The diagnosis was legibly written in 842 (69.5%), while the patient weight was recorded in 307 (25.3%) prescriptions. The least commonly recorded instruction was the drug strength (26.8%), while the dose and frequency of use were stated for 91.3% and 90.8% of the drugs, respectively. The average number of drugs per prescription was 2.35 ± 0.89; 72.97% drugs were prescribed by generic name; in 65.98% patient encounters, antibiotics were prescribed. The overall IRDP was 3.56. Most of the physicians and pharmacists reported availability of the drug list and Saudi PHC formulary in their PHCCs.

**Conclusion:**

PHC physicians’ drug prescribing was not at the optimal level of rational use, especially regarding prescription of antibiotics. Creating awareness about rational drug use and hazards of overuse of antibiotics is needed.

## Introduction

Children comprise a large proportion of the population, especially in developing countries. Morbidity in this vulnerable group is usually high. Hence, they need special attention for their health care. This age group is more vulnerable to the harmful effects of medicines. However, irrational prescription of medicines in this group of patients is reported to be widespread (Hogerzeil, 1995). In various studies, irrational and excessive use of antibiotics has been documented among pediatric patients (Oshikoya *et al*., 2009). Irrational antibiotic use can lead to antimicrobial resistance, treatment failures, and increased healthcare costs (Hersh *et al*., 2013; Fadare *et al*., 2015). Polypharmacy and other forms of inappropriate prescribing can be extremely harmful in children (Fadare *et al*., 2015). Irrational prescribing may also result in adverse drug reactions (Oshikoya and Ojo, 2007). Thus, it is important to study the prescribing pattern of physicians in pediatric population (Cole *et al*., 2015).

To assess the prescribing practices in healthcare facilities, World Health Organization (WHO) developed a set of standardized drug use indicators (WHO, 1993). These prescribing indicators are useful in identifying problems in general prescribing. Using these WHO indicators, various studies have been conducted to assess the prescribing patterns for children in different parts of the world (Nwolisa *et al*., 2006; Oshikoya *et al.*, 2006; Akhtar *et al*., 2012).

Saudi Arabia has an estimated population of 31.8 million people in 2016, out of which 20.1 million are Saudis (General Authority of Statistics, 2016). Saudi children below five years constitute 10.6% of the Saudi population (General Authority of Statistics, 2016). In Saudi Arabia, children aged five years and below constitute the bulk of patients attending the primary health care centers (PHCCs). Generally, there is inadequate data on prescribing in the Arabian Gulf countries (Khoja *et al*., 2011). In Saudi Arabia, a few studies conducted on prescription pattern have mostly focused on the hospital setting (Al-Abbassi and Madani, 1987; al-Nasser, 1991; Bawazir, 1993; Al-Dawood, 1995; Khoja *et al*., 1996; Balbaid and Al-Dawood, 1998; Qureshi *et al*., 2001; Neyaz *et al*., 2011). However, the greater burden of health care and prescription of drugs is shared by the primary health care (PHC) system (Neyaz *et al*., 2011). There is variation in prescribing patterns at various levels of health care (Neyaz *et al*., 2011). These variations exist between various countries as well as among various regions of the same country (Neyaz *et al*., 2011). More variations in prescriptions are expected because of certain unique features of the healthcare system in Saudi Arabia, including a large number of expatriates working as PHC physicians (Neyaz *et al*., 2011), underscoring the importance of studying the prescription patterns in the PHC system.

In Saudi Arabia, a few prescription audits have been conducted but mainly among adult patients. On literature search, we were not able to find studies on the pediatric prescribing pattern at the PHC level. To address the gap, we designed this study to assess the prescribing patterns for children under five years at the PHCCs in Qassim using the WHO recommended prescribing indicators for drug use. Our study will be helpful in developing interventions to enhance the quality of prescribing for children less than five years old. The purpose of this study is to determine the physicians’ drug prescribing practices for children aged below five years at the PHCCs in Qassim. Our study also determines the completeness of the recorded information on the prescriptions, and analyzes the core indicators of drug prescribing at the PHCCs in Qassim.

## Methods

Using the WHO model of drug utilization study (WHO, 1993), this cross-sectional study of medication prescriptions was conducted for selected PHC centers in Qassim. Moreover, the physicians and pharmacists working in the selected centers were also surveyed, to collect relevant information.

Qassim is served by 177 PHC centers (Ministry of Health, 2016). These centers offer services to almost homogeneous population. To comply with the WHO standard of 20 health centers to conduct such studies (WHO, 1993), we selected 25 PHC centers by systematic random sampling. All prescriptions, for the first week of first six Hijrah months from Moharram to Jamadi II of the year 1437 Hijrah (October 2015 to April 2016), were procured from the selected PHCCs. All received prescriptions were reviewed and organized according to the dates of the treatment and age of the patient. The prescriptions for patients with more than 5 years old were excluded. Moreover, prescriptions from the dental clinic as well as the prescriptions written for childhood vaccination were excluded.

A data collection form was designed to record information from the prescriptions. The form was pretested and was modified accordingly. The patient demographics, diagnoses, and medications on each prescription were recorded on the data collection form. The number of drugs per prescription, strength and dose of the drug, and frequency and duration of administration were recorded. Moreover, use of generic names, antibiotics, and injections were also recorded. The number of drugs prescribed from Saudi primary health care (SPHC) formulary, prescribing physician’s name and signature with stamp, were noted. The prescriptions were analyzed for completeness and basic drug use indicators.

WHO specifies (WHO, 1993) the following drug use indicators that were used in the study to determine the prescribing pattern:

Average number of drugs per encounter (measures the degree of polypharmacy)

- Percentage of drugs prescribed by a generic name (measures the cost-effectiveness of a health system to procure and use drugs)
- Percentage of encounters with an antibiotic prescribed (measures the level of use of commonly overused and expensive form of drug therapy)
- Percentage of encounters with an injection prescribed (measures the level of use of commonly overused and expensive form of drug therapy)
- Percentage of drugs prescribed from the national essential medicines list.

### Index of rational drug prescribing

The index of rational drug prescribing (IRDP) consists of five indices derived from the WHO drug prescribing indicators (Dong *et al*., 2011). Table 1 displays the optimal level for each indicator. Each indicator has an optimal index of 1. Drug prescribing is considered more rational for the values of calculated index closer to 1. In our study, the prescriptions with three or more medicines were considered as polypharmacy. The generic name index and essential medicine index were measured by the percentage of drugs prescribed by generic name and from the SPHC formulary, respectively. The index of rational antibiotic prescribing was defined as dividing the optimal level (30%) by the percentage of prescriptions including an antibiotic. The index of prescribing injection was calculated by dividing the optimal level (10%) by the percentage of prescriptions including the injection. The IRDP with a maximum value of 5 was calculated by adding the indices.

**Table 1.** Optimal levels of drug prescribing indicators

Prescribing indicators	Optimal level (%)	Optimal index
Percentage of polypharmacy prescriptions	≤3	1
Percentage of drugs prescribed by generics	100	1
Percentage of prescriptions including antibiotics	≤30	1
Percentage of prescriptions including injections	≤10	1
Percentage of drugs prescribed from formulary	100	1

Adapted from Cole *et al*. (2015)

We screened 25,012 prescriptions for the first five days of the first six months of the year 1437 Hijrah, received from 25 PHC centers. There were 4125 (16.5%) prescriptions for children under five years. For each center, the prescriptions of all patients under five years old were arranged according to dates and were numbered. Fifty prescriptions were randomly selected from each center. For the centers where prescriptions were 50 or less in number, all prescriptions were included in the study. Thus, 1212 prescriptions were sampled for the study. A copy of the original prescription was used for the data extraction, and the data were entered on the data collection form.

In addition to prescriptions, data were also collected from physicians and pharmacists. All data for the project were collected during June–July 2016. Two different questionnaires were designed to gather information from the physicians and the pharmacists. An informed consent along with the questionnaire was sent by fax to all physicians of the selected PHCCs. All physicians working in the sampled PHCCs were included in the survey. However, the physicians working for less than a month and the dentists were excluded from the study. The physicians of the selected PHCCs completed a self-administered questionnaire. The questionnaire was designed to gather information about basic demographic data of the physicians and information about prescription writing.

One pharmacist working in each sampled PHCC was interviewed via telephone. The pharmacist available at the time of telephone call was interviewed. In the case of non-availability of the pharmacists, the persons performing their duties for more than a month were interviewed. A short interviewer-administered questionnaire was designed to gather information about the number of assigned and available pharmacists in the PHCC, as well as the availability of drug list and SPHC formulary. The pharmacist of each of the selected PHCC was informed regarding the nature of the study, and the interview was conducted via telephone.

The data were entered and analyzed using Epi Info version 3.5.4. Results were expressed as means, frequencies, and percentages. Relevant inferential statistical tests were used to determine the level of significance with *P* values <0.05 considered significant.

The confidentiality of the prescriptions was assured. No names were entered in the database, and each prescription was given a unique identifier number for the handling of data. Furthermore, ethical approval was obtained from the Regional Research Ethics Committee, Qassim province.

## Results

A total of 25 PHCCs were sampled for this study. Mean population in the catchment area of these PHCCs was 4955 persons with a minimum of 829 and a maximum of 12,412 persons per PHCC. The number of assigned physicians ranged from one to five, with approximately one-third (32%) of the centers having two physicians assigned.

A total of 25 respondents (one person from each center) responded to the telephone interview for pharmacists. Most (72%) of the centers had pharmacist dispensing the medicines; however, in seven centers (28%), a nurse was assigned to dispense the medicines. According to the respondents, the drug list was available in all PHCCs; 20 centers (80%) were using drug list issued in the current year while five centers (20%) had previous year’s drug list. A total of 16 PHCCs (64%) had received SPHC formulary. However, the pharmacists reported varied years of publication for the SPHC formulary.

At the time of the physicians’ survey, 59 physicians were assigned to the sampled 25 PHCCs. Forty-eight physicians (81.4%) responded to the survey. All non-respondent physicians were on vacations at the time of the survey. The mean age of the physicians was 39.7 (±9.7) years, median years since graduation were 15 with a minimum of 1 year and a maximum of 36 years while the median years of experience in PHCCs were five years with a minimum of 4 months and a maximum of 30 years. Most (62.5%) of the physicians had a degree of Bachelor of Medicine; however, 18 (37.5%) physicians had a postgraduate qualification. Table 2 displays the salient findings of the physician’s survey. Most (79.2%) of the physicians responded that after their graduation, they had never attended any refresher course or training about prescription writing.

**Table 2.** Findings of physicians’ survey at sampled PHCCs

Gender (*n* = 48)	No. (%)
Male	28 (58.3)
Female	20 (41.7)
**Nationality (** ***n*** ** = 47)**	
Non-Saudi	44 (93.6)
Saudi	3 (6.4)
**Responses to prescription-related questions**	
Were taught for prescription writing during academic training (*n* = 48)	35 (72.9)
Had attended course for prescription writing after graduation (*n* = 48)	10 (20.8)
Saudi PHC formulary available at PHCC (*n* = 48)	41(85.4)
Saudi PHC formulary available in physician’s clinic (*n* = 46)	25 (54.3)
Drug list available at PHCC (*n* = 47)	45 (95.7)
Drug list available in physician’s clinic (*n* = 45)	39 (86.7)

### Prescriptions data

In our study, 1212 prescriptions were analyzed for completeness of basic information of the patients as well as WHO drug indicators.

### Demographic data of patients

The mean age of the patients in our study was 2.62 (±1.52) years. There was almost an equal gender distribution with slightly more male patients (51.75%). Most of the patients were Saudis comprising 95.9% of the total patients attending PHCCs. The distribution of diagnoses recorded on the sampled prescriptions is displayed in Table 3.

**Table 3.** Distribution of diagnoses recorded on prescriptions from PHC centers

**Diagnosis recorded**	Prescriptions from PHC centers (*n*= 842)	
	No.	Percent*
Upper respiratory tract infection	645	76.6
Lower respiratory tract infection	60	7.1
Gastrointestinal disorder	57	6.8
Skin disease	23	2.7
Eye disease	13	1.5
Miscellaneous infections	11	1.3
Trauma	9	1.1
Ear disease	7	0.8
Other	17	2.0
**Total**	**842**	**˜100.00%**

* *The percentage does not add up to 100% due to rounding off*.

### Basic information recorded on prescriptions

The completeness of most of the basic information including date, patient age, and gender was more than 90% (Table 4). The physicians signed most (95%) of the prescriptions. The weight of the patients was the least recorded information with only 307 (25.3%) prescriptions with patients’ weight written on them (Table 4).

**Table 4.** Distribution of information recorded on prescriptions from PHC centers

Information recorded	Prescriptions from PHC centers (*n* = 1212)	
	No.	Percent
Date	1193	98.4
File number (including ‘No file’ recorded)	1074	88.6
Patient’s gender	1140	94.1
Patient’s nationality	1076	88.8
Patient’s weight	307	25.3
Patient’s age	1198	98.8
Diagnosis code	1059	89.4
Diagnosis		
Recorded	842	69.5
Not recorded	329	27.2
Not readable	41	3.4
Physician name	936	77.23
Physician signature	1151	95.0
Stamp	802	66.2
**Distribution of information recorded as instructions for drugs**		
Strength stated (*n* = 1859) ^¶^	498	26.8
Frequency stated (*n* = 2290)	2079	90.8
Dose stated (*n* = 2132) *	1947	91.3
Duration of drug use stated (*n* = 2290)	1178	51.4

¶ Combination drugs excluded

* Creams and ointments excluded

### Prescribing indicators

Table 5 summarizes the drug prescribing indicators at the PHCCs. The average number of drugs per prescription was 2.35 ± 0.89 (minimum 1 and maximum 6). Prescriptions for more than three drugs were 97 (8.01%).

**Table 5.** Drug prescribing indicators at the PHC centers

Drug prescribing indicator	Average/Percentage	Standards
Average number of medicines prescribed per encounter	2.35 ± 0.89	
Percentage of polypharmacy (more than three drugs) prescriptions	97 (8.01%)	**≤3**
Percentage of drugs prescribed by generic name (combination drugs excluded)	72.97%	**100%**
Percentage of encounters with an antibiotic prescribed	799 (65.98%)	**≤30%**
Percentage of encounters with an injection prescribed	8 (0.66%)	**≤10%**
Percentage of medicines prescribed from the SPHC formulary	100.00%	**100%**

The core drug indicators were also calculated individually for each of the 25 PHCCs. The maximum mean number of drugs was 2.84 (±1.24) while the minimum was 1.90 (±0.86). The maximum proportion of antibiotic prescriptions was 88.37% while the minimum was 22%, with only one center having the antibiotic prescription of less than 30% cut-off point according to WHO criteria. The prescription of drugs by generic name varied among the PHC centers. It ranged from 28.8% as the minimum to 98.98% as the maximum proportion of drugs prescribed by generic name. All centers met the criteria for prescription of injections and the proportion of drugs prescribed from SPHC formulary.

### The index of rational drug prescribing

The overall IRDP used as an indicator of rational drug use was 3.56 with the optimal level of 5. The overall IRDP 3.56 was made up of the index of antibiotic (0.45), polypharmacy (0.38), injection (1.00), generic name (0.73), and drugs prescribed from the SPHC formulary (1.00) (Table 6).

**Table 6.** Index of rational drug prescribing (IRDP)

Prescribing indicators	Optimal level (%)	Optimal index	Level (%) calculated	IRDP
Percentage of polypharmacy (more than three drugs) prescriptions	**≤ 3**	**1**	97 (8.01%)	**0.38**
Percentage of drugs prescribed by generic name	**100**	**1**	72.97%	**0.73**
Percentage of prescriptions including antibiotics	**≤30**	**1**	799 (65.98%)	**0.45**
Percentage of prescriptions including injections	**≤10**	**1**	8 (0.66%)	**1**
Percentage of drugs prescribed from SPHC formulary	**100**	**1**	100.00%	**1**

The index of rational drug prescribing was also calculated for each PHCC individually. The highest IRDP was 4.89, while the lowest was 2.75. All PHCCs had an index of 1 for injection prescription and prescription of drugs from the SPHC formulary. The indices of polypharmacy, prescription of antibiotics, and prescription of drugs by generic names varied among the PHCCs. The highest index for polypharmacy was 1.00, while the lowest was 0.10. The highest index for prescription of antibiotics was 1.00, while the lowest was 0.34. The index for prescription of drugs by generic name also varied with a maximum score of 0.99 while it varied with a minimum score of 0.29.

## Discussion

Our study explored the drug-prescribing pattern of physicians and the completeness of recorded information on the prescriptions written in PHC centers.

### Prescribing indicators

In our study, the average number of drugs per prescription was 2.35 ± 0.89, which is similar to other studies reporting the average number of drugs per prescription as 2.82 ± 1.3 (Atif *et al*., 2016), 2.2 ± 0.8 (Bilal *et al*., 2016), 2.6 ± 1.1 (Fadare *et al*., 2015), 2.4 ± 1.2 (El Mahalli, 2012), and 2.55 (Yousif and Supakankunti, 2016). In contrast, some studies have reported higher average number of medicines per prescription as 3.77 (Cole *et al*., 2015) and 3.3 ± 0.7 (Otoom etal., 2010), whereas others have reported lower mean number (1.92) of drugs per prescription (Fattouh and Abu Hamad, 2010).

Prescription writing using generic names is important for better communication among healthcare workers (Cole *et al*., 2015). The percentage of drugs prescribed by generic name (72.97%) in our study are similar to those reported by other studies: 64.12% (Dong *et al*., 2011), 84% (Kasabi *et al*., 2015), 71% (Cole *et al*., 2015), and 68.9% (Fadare *et al*., 2015). In contrast, some studies have reported lower percentage, such as 56.6% (Atif *et al*., 2016), 46.34% (Yousif and Supakankunti, 2016), 10.2% (Otoom *et al*., 2010), and 7.4% (Pandey *et al*., 2010). A study conducted among PHCCs in Saudi Arabia reported variable percentage of drugs prescribed by generic name ranging from 6.0% to 99.9% (El Mahalli, 2012). Another study reported the very high use of generic names (97%) for prescribed drugs (Bilal *et al*., 2016).

An inappropriate use of antibiotics, resulting in the development of resistance, causes increased morbidity as well as elevated healthcare costs (Cole *et al*., 2015). In our study, the percentage of prescriptions with antibiotics was 65.98%, which is much higher than the WHO recommended cut-off of 30%. Other studies have also reported over-prescription of antibiotics including 82.5% (Bilal *et al*., 2016), 74.8% (Cole *et al*., 2015), and 71.1% (Fadare *et al*., 2015), of the patient encounters. Some studies have reported lower prescription of antibiotics as compared to our study: 49% (Kasabi *et al*., 2015), 51.5% (Atif *et al*., 2016), 54.71% (Yousif and Supakankunti, 2016), and 44.6% (Sharif *et al*., 2015) of the patient encounters. However, most of these studies are conducted on adult population.

Inappropriate use of injections can have harmful consequences to the patients. In our study, the percentage of encounters with prescription of an injection (0.66%) was very low as compared to those reported by other studies: 61% (Kasabi etal., 2015), 22.93% (Dong *et al*., 2011), and 21.1% (Cole *et al*., 2015). In contrast, some studies reported the very low proportion of prescriptions with injections as 1.6% (Pandey *et al*., 2010) and 0.38% (Sharif *et al*., 2015), while Atif *et al*. (2016) reported no injection prescription.

Prescribing drugs from National Essential Drug List is an important drug use indicator. In our study, all drugs (100%) were prescribed from the SPHC formulary. The reason for this 100% achievement is that the PHCCs are provided all required medicines by Saudi Ministry of Health, and the physicians are bound to prescribe medicines from the officially available drugs. Other studies have also reported high percentage of drugs prescribed from their National Essential Drug List: 100% (Sharif *et al*., 2015), 99.2% (El Mahalli, 2012), 98.8 % (Atif *et al*., 2016), 94% (Kasabi *et al*., 2015), and 92% (Bilal *et al*., 2016). In contrast, some studies have reported lower proportion of prescriptions using National Essential Drug List: 81.19% (Yousif and Supakankunti, 2016), 70.6% (Cole *et al*., 2015), 67.70% (Dong *et al*., 2011), 60.4% (Fadare *et al*., 2015), and 38.9% (Pandey *et al*., 2010).

### The index of rational drug prescribing

In our study, the overall IRDP used as an indicator of rational drug use was 3.56 with the optimal level of 5. It is in comparison with other studies reporting similar IRDP: 3.32 (Dong *et al*., 2011), and 3.42 (Kasabi *et al*., 2015). In contrast, some studies have reported lower (2.71) IRDP (Cole *et al*., 2015). In our study, the index of antibiotic was 0.45 compared to an index of antibiotic of 0.62 (Dong e*t al*., 2011), 0.68 (Kasabi *et al*., 2015), and 0.40 (Cole *et al*., 2015) reported by other studies.

## Prescriptions data

### Basic information recorded on prescriptions

For patient management and administrative purposes, each prescription should have patient’s demographic information recorded on it. The comparison of results of our study with a similar study conducted in Riyadh, Saudi Arabia showed that the recorded information was better in our study as compared to Neyaz etal. (2011) for date (67.3%), file number (60.6%), nationality (80.5%), age (89.3%), and diagnosis code (80.9%). However, patient gender (100%), diagnosis (91%), and physician’s signature (96.8%) were better recorded in the study by Neyaz et al (2011).

In our study, the most frequent diagnosis recorded in prescriptions was upper respiratory tract infection that corresponds with another study conducted among PHCCs in Saudi Arabia in which 51.2% of the diseases recorded on prescriptions were upper respiratory tract infections (Neyaz *et al*., 2011). Another study reported acute respiratory tract infections (53.7%) as the most common diagnoses among children at the outpatient clinic (Fadare *et al*., 2015).

### Drug-related information recorded on prescriptions

Writing proper information on the prescriptions, including the strength and dose of the drug, frequency, and duration of use of drugs, is vital for clear instructions to the pharmacists and appropriate management of the patients. Neyaz *et al*. (2011) reported poor quality of instructions provided to the pharmacists. According to the study, the strength of the drug was noted for only 17.3% of prescriptions that are comparable to 26.8% of the prescriptions in our study. The details of the drug dose were better recorded (76.3%–80.6%) in this study, similar to our study where drug dose was stated for 91.3% drugs. The highest proportion reported for the frequency of drug use in Neyaz study was 81.5% while our study reported it to be stated for 90.8% of the medicines. In our study, duration of drug use was recorded for only half (51.4%) of the drugs prescribed in comparison to the highest 66.2% reported in the study by Neyaz *et al*. (2011). Other studies have also found incompletely recorded basic drug information (Al Khaja *et al*., 2005). One study reported that only 25% of the medicines dispensed were adequately labeled with a mention of strength, dosage, and duration (Kasabi *et al*., 2015). The incompleteness of the prescriptions may be explained by various factors such as crowded outpatients at PHCCs leading to hurried prescription writing. Lack of awareness of the importance of the completeness of the recorded information may be another factor, underscoring the importance of creating awareness among the PHCC physicians about this important issue. Moreover, an effective monitoring system for completeness of prescriptions may be put in place to improve the quality of prescriptions.

In the sampled PHCCs, we also gathered information from physicians and pharmacists, regarding prescription writing. The demographic data of the participant physicians in our study were similar to another study (Magzoub *et al*., 2011) conducted in Saudi Arabia where the proportions of male physicians (62%) and postgraduate qualification (31%) were close to the proportions in our study. In contrast to the study by Magzoub and colleagues (2011), where only one-third (35%) of the physicians reported of receiving the training in prescription writing at their medical schools, in our study almost three-fourths (73%) reported being trained in prescription writing during their academic training. In our study, 95.7% physicians and 100% pharmacists were aware of the availability of drug list in their PHCCs compared to another study where 85% physicians and 75% pharmacists were aware of essential medicines list (Kasabi etal., 2015).

### Limitations

Our study had certain limitations. It was conducted in PHCCs only and does not include prescribing indicators from the hospitals. Prescription of drugs not included in Ministry of Health drug list that may be advised for purchase from private pharmacies using an extra prescription could not be assessed. The study does not explore the reasons for irrational drug prescribing, especially with regards to antibiotics. Reasons for irrational use of antibiotics may be explored by future research. The study was conducted in only one province of Saudi Arabia; hence, it may not be generalizable to other provinces.

## Conclusion

Our study demonstrates that PHC physicians’ drug prescribing was not at the optimal level of rational use, especially with regards to the prescription of antibiotics. Over-prescription of antibiotics to children is a major issue among physicians in the PHCCs, underscoring the importance of creating awareness about hazards of over-prescribing antibiotics generally and especially to the children under five years.

## Implications for policy and practice

There is a need to develop training programs focusing on PHC physicians to encourage them to comply with national and international standards in prescription writing and to address the issue of the rational use of drugs, especially rationale use of antibiotics. We also recommend the provision of continuous on-job training for prescription writing for PHC physicians and pharmacists, and regular evaluation of prescriptions.
